# Local structure of liquid gallium under pressure

**DOI:** 10.1038/s41598-017-05985-8

**Published:** 2017-07-18

**Authors:** Renfeng Li, Luhong Wang, Liangliang Li, Tony Yu, Haiyan Zhao, Karena W. Chapman, Yanbin Wang, Mark L. Rivers, Peter J. Chupas, Ho-kwang Mao, Haozhe Liu

**Affiliations:** 1 Harbin Institute of Technology, Harbin, 150080 China; 2 Center for High Pressure Science and Technology Advanced Research, Changchun, 130015, Beijing, 100094 China; 3 X-ray Science Division, Advanced Photon Source, Argonne National Laboratory, Argonne, Illinois 60439 USA; 4 Center for Advanced Radiation Sources, The University of Chicago, Chicago, Illinois 60637 USA; 5 Center for Advanced Energy Studies, University of Idaho, Idaho Falls, Idaho 83406 USA; 6 Geophysical Laboratory, Carnegie Institution, Washington, DC 20015 USA

## Abstract

*In situ* high energy X-ray pair distribution function (PDF) measurements, microtomography and reverse Monte Carlo simulations were used to characterize the local structure of liquid gallium up to 1.9 GPa. This pressure range includes the well-known solid-solid phase transition from Ga-I to Ga-II at low temperature. In term of previous research, the local structure of liquid gallium within this domain was suggested a mixture of two local structures, Ga I and Ga II, based on fitting experimental PDF to known crystal structure, with a controversy. However, our result shows a distinctly different result that the local structure of liquid gallium resembles the atomic arrangement of both gallium phase II and III (the high pressure crystalline phase). A melting mechanism is proposed for Ga, in which the atomic structure of phase Ι breaks up at the onset of melting, providing sufficient free volume for atoms to rearrange, to form the melt.

## Introduction

The absence of long-range atomic order in the liquid state makes it challenging to characterize liquid structure. Short-range atomic order, within a few Å, can be captured with local structure probes, such as pair distribution function (PDF)^1, 2^. More extended models for the atomic structure can be derived from three-dimensional configurations such as those generated through reverse Monte Carlo (RMC) modeling, whereby the PDF calculated for a large atomic configuration is optimized against the experimental PDF^3, 4^. Application of RMC analysis requires that the sample density should be known. This is challenging for liquids under variable pressure conditions. Here, synchrotron X-ray microtomography is valuable; the density of noncrystalline materials under extreme conditions can be directly quantified^5, 6^.

Understanding the local structure of stable and undercooled melts is important to understand crystallization and glass formation from the melt and the properties of the liquid itself^7^. A growing body of research has demonstrated that although icosahedral short-range ordering dominates stable and undercooled transition metal liquids, the structures also contain local regions with varying degrees of fcc, hcp or bcc order^7–15^. For gallium, which exhibits a rich polymorphism in the solid state^16–22^, the local structure of the melt under pressure is not yet well understood. A first-principle study suggests the local structure of liquid gallium is similar to Ga-II or Ga-III^23^. Subsequently, an X-ray scattering study, showing a different result, proposes that its local structure is similar to the mixture of two local structures, Ga I-like and Ga II-like^24^. To shed light on this contradiction, the local structure of liquid gallium is studied under high pressure at ambient temperature using PDF analysis in combination with RMC simulation, constraining the densities to those quantified through measurement by X-ray microtomography.

## Results and Discussion

**Figure 1:**
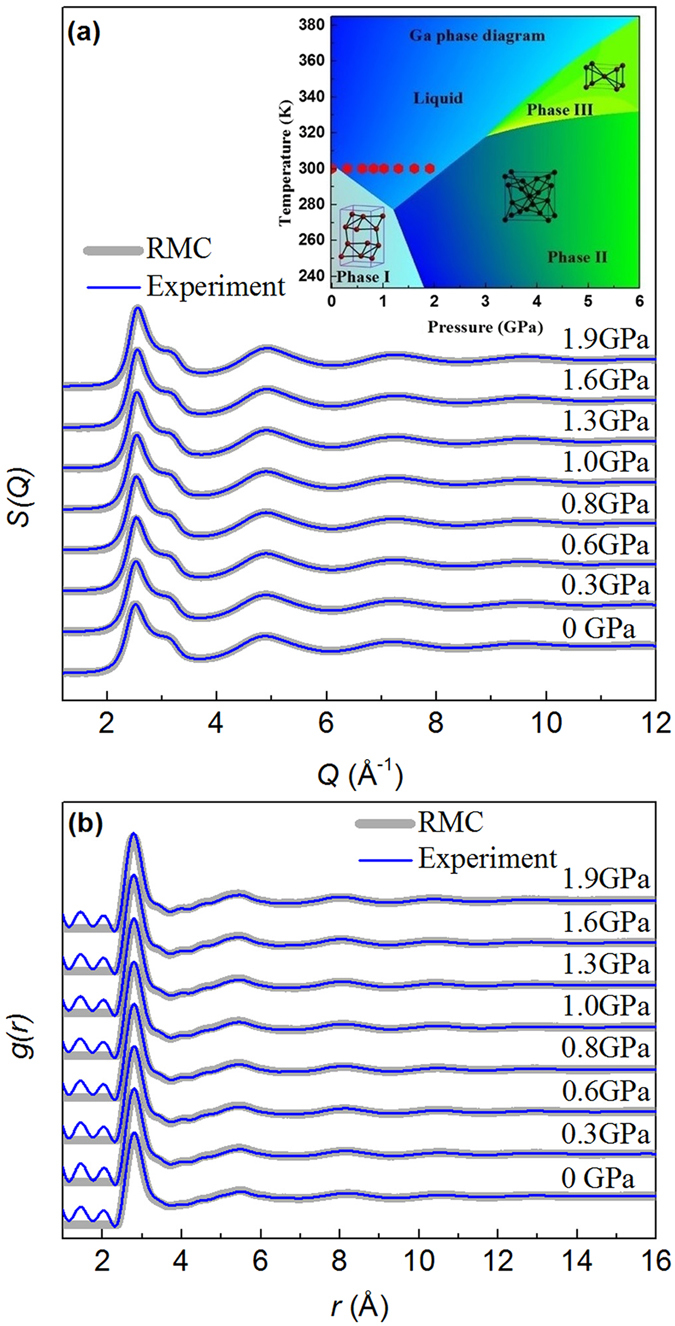
(**a**) Structure factors *S*(*Q*) and (**b**) pair distribution function *g*(*r*) from experiments (blue thin line) at various pressure conditions and corresponding RMC simulations (gray thick line). Crystalline structure of Ga phase I, II and III are illustrated in their corresponding phase domains. The red hexagon represents the experimental data in this work.

**Figure 2:**
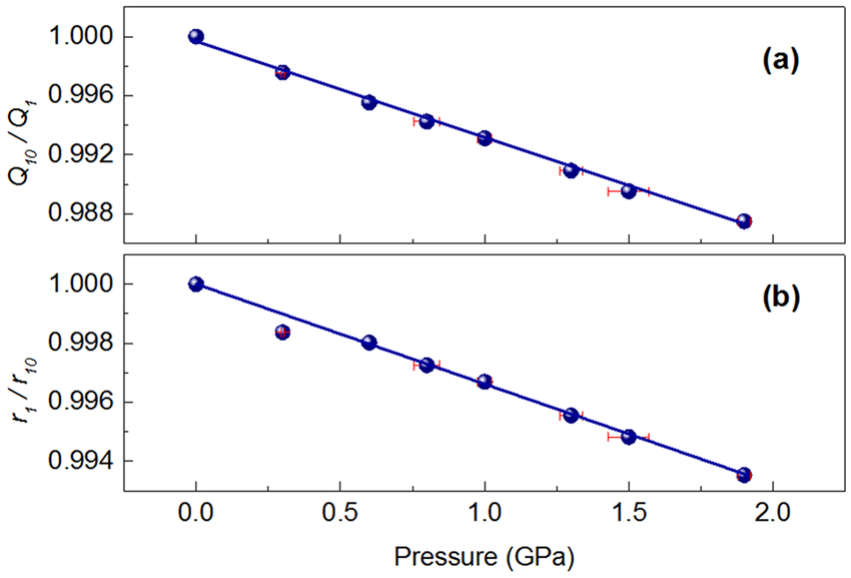
(**a**) The normalized first peak position *Q*_*1*_ of the structure factor *S*(*Q*) and (**b**) nearest-neighbor distance *r*_*1*_ as a function of pressure. *Q*_*10*_ and *r*_*10*_ are the initial first peak positions in reciprocal and real space, respectively.

The experimental structure factor *S*(*Q*), PDFs, and the corresponding RMC simulation results at various pressure conditions are shown in Fig. 1. The RMC simulations provide a good fit to the experimental data at each pressure point, and hence, can provide valuable insights into the atomic structure. Figure 2(a) and (b) show the normalized first peak position *Q*_*1*_ of the structure factor *S*(*Q*) and nearest-neighbor distance *r*_*1*_ as a function of pressure. As expected due to densification under pressure, *Q*_*1*_ shifts towards higher *Q* values and *r*_*1*_ shifts to shorter distances with increasing pressure. Both the first peak position *Q*_*1*_ and the nearest-neighbor distance *r*_*1*_ change linearly as a function of pressure, suggesting that no detectable liquid-liquid phase transition occurs in this pressure range, which is consistent with previous studies^25, 26^.

To characterize the local atomic environment of liquid gallium, the Voronoi tessellation method^27, 28^ was employed, by which information on the coordination number (CN) could be determined as well. The Voronoi polyhedron is indexed by <n_3_, n_4_, n_5_, n_6_ …>, where n_i_ denotes the number of i-edged faces of the Voronoi polyhedron, to specify the type of polyhedron describing the neighborhood of the associated central atom. In constructing the Voronoi polyhedra, surfaces with area contributions <1% of the total surface area were ignored to minimize the degeneracy problem and the effects of thermal vibration^29^.

**Figure 3:**
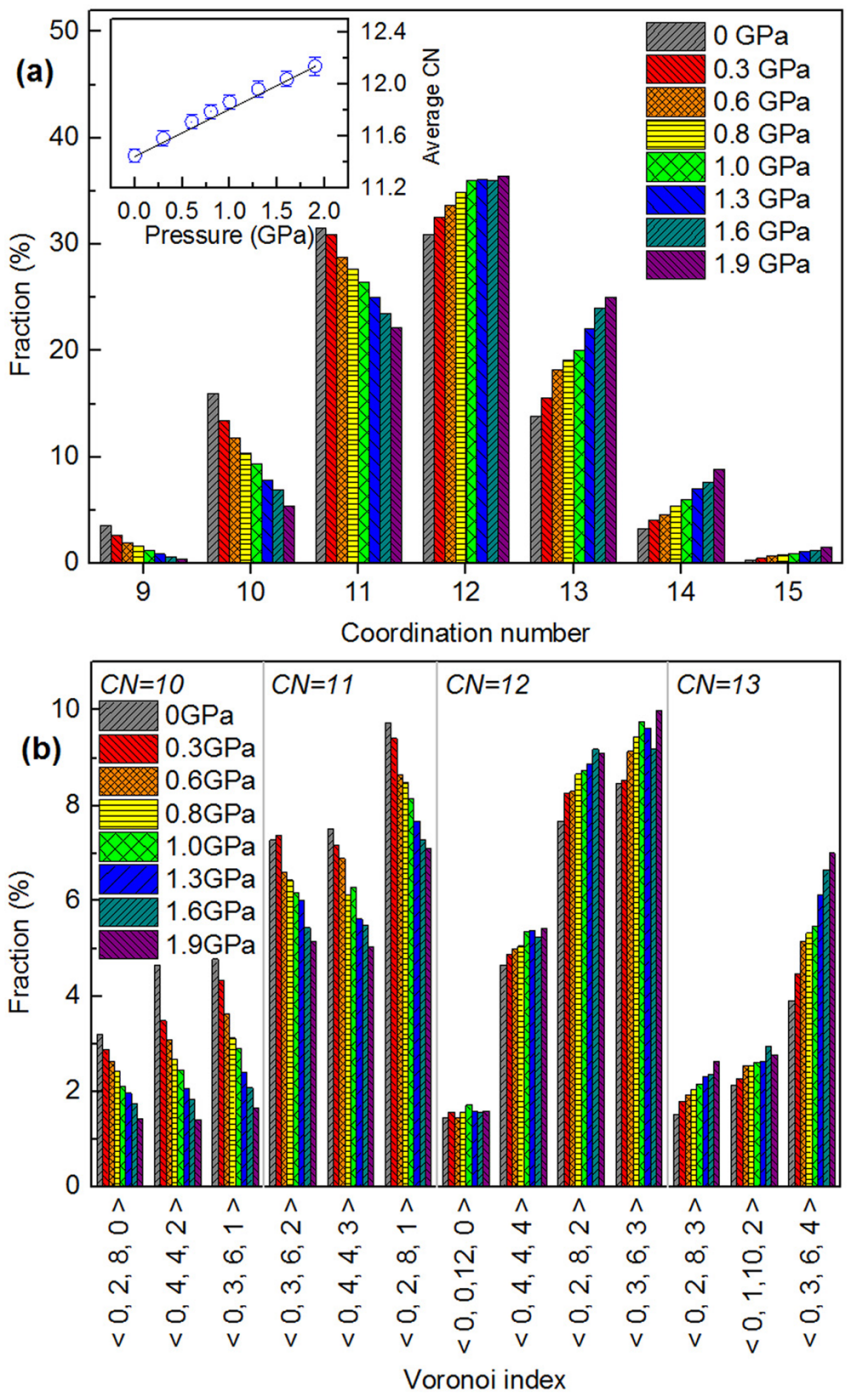
(**a**) The distribution of the CN for liquid gallium at various pressure conditions. The inset illustrates average CN as a function of pressure. (**b**) Fractions of dominant coordination polyhedra at different pressures. Note that only polyhedra with a fraction of >1.5% are shown.

Figure 3(a) shows the distributions of the CNs for liquid gallium from the atomic configurations generated through RMC. The inset of Fig. 3(a) shows the average CN as a function of pressure, which suggests that liquid gallium has an average CN of 11.4 at ambient conditions. This is a typical value for liquid metals with a conventional close-packed structure. For example, liquid aluminum, indium and thallium have an average CN of 11.5, 11.6 and 11.6, respectively^30^. The value of the average CN for liquid gallium increases gradually from 11.4 to 12.1 with pressure increasing from 0 to 1.9 GPa. These values are comparable to those of 11.7, 12.1 and 12.2 at 0, 1.6 and 2.5 GPa, respectively, reported in literature^23^. The most common CNs, 11, 12 and 13, account for about 81% of the Ga coordination environments at these pressure conditions.

Figure 3(b) shows the frequency of the dominant Voronoi polyhedron types corresponding to various CNs derived from RMC simulations. Note that there is a one-to-one correspondence between the Voronoi index and the coordination polyhedra. Voronoi polyhedra with indices <0, 2, 8, 0>, <0, 3, 6, 4> and <0, 0, 12, 0> correspond to the Archimedean prism, deformed crystal and icosahedron polyhedra, respectively. These are present in small fractions in liquid Ga. Polyhedra of deformed prisms, with indices <0, 3, 6, 1>, <0, 4, 4, 2> and <0, 2, 8, 1>, contribute to 28.6% and 16.8% of high frequency polyhedron types in liquid gallium at 0 and 1.9 GPa, respectively. In contrast, deformed icosahedron polyhedra indexed by <0, 3, 6, 2>, <0, 4, 4, 3>, <0, 4, 4, 4>, <0, 2, 8, 2>, <0, 3, 6, 3>, <0, 2, 8, 3> and <0, 1, 10, 2> appear with high frequency for liquid gallium, amounting to 58.6–66.7% of the polyhedra at pressures from 0 to 1.9 GPa. Thus, deformed icosahedron polyhedron type ordering is the dominant topological order for liquid gallium under pressure.

To examine the correlation in local atomic motifs between liquid gallium and related crystalline phases, Voronoi polyhedra derived from Ga-I^31^ and Ga-II^18^ were compared with those for liquid gallium at the same number density. The proportion of <0, 4, 4, 1> polyhedron type, which corresponds to the index of short range order extracted from Ga phase I at 3.71 Å cutoff, is 0.8% at 0 GPa and negligible at 1.9 GPa. In contrast, the dominant Voronoi polyhedra corresponding to CN of 12 including <0, 4, 4, 4>, <0, 2, 8, 2> and <0, 3, 6, 3> appear in the structure of Ga-II, where <0, 4, 4, 4> also presents in Ga-III at the same cutoff. Ga-II has a body-centered cubic (bcc) structure, while Ga-III has a body-centered tetragonal (bct) cell^18^. The bct can be viewed as a distortion of bcc^32^. Furthermore, Ga-II is considered as a precursor of Ga-III^23^, hence they sharing the same motifs is reasonable. The Voronoi polyhedra analyses suggests that the local structure of liquid gallium at room temperature is more similar to the local atomic structure of the high pressure solid phase both Ga-II and Ga-III among its deformed icosahedra within the range relevant to high pressure melting.

**Figure 4:**
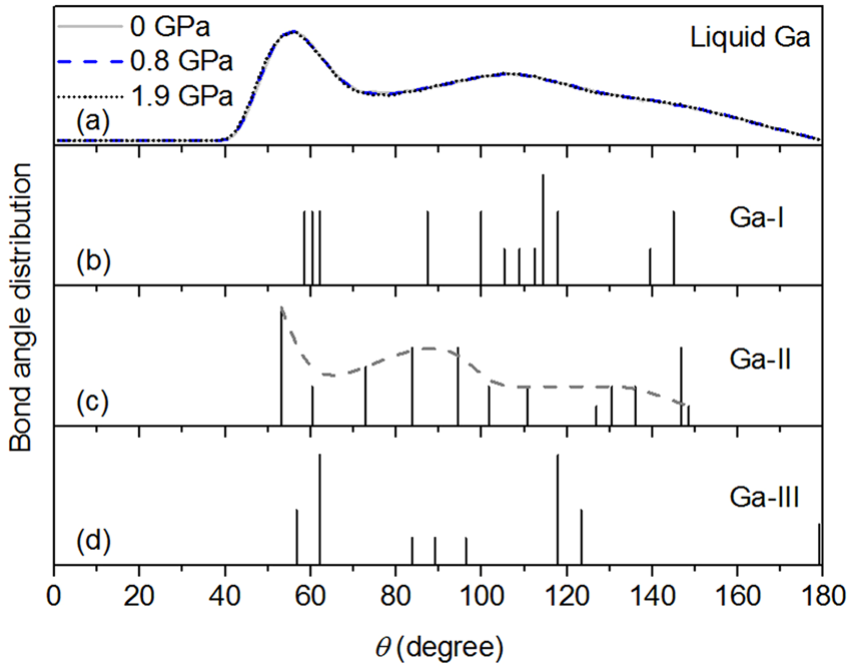
The bond angle distribution in (**a**) liquid gallium, (**b**) Ga phase I, (**c**) Ga phase II and (**d**) Ga phase III. The gray dashed curve is the profile from connecting all the peaks of bond angle distribution in Ga phase II.

The bond angle distribution is often used to describe topological short range ordering in liquid and amorphous structures. The distribution of bond angles defined by three atoms within a cutoff distance of 3.71 Å, at selected pressures, are shown in Fig. 4(a). This shows that the pressure dependence of the bond angle distribution in liquid gallium is not large. A prominent peak and a broad maximum are located at *θ*~56° and *θ*~106°, respectively. The comparison of bond angle distribution in liquid gallium with Ga-I^31^, Ga-II and Ga-III^18^ are shown in Fig. 4. The angle distribution of Ga-I displays a dominant peak at 114°, which is away from that of liquid gallium. In contrast, both Ga-II and Ga-III present the predominant angle distribution between 53° and 62°, similar to that of liquid gallium. Considering the angle distribution of liquid gallium from 70° to 180°, it resembles a mixture of Ga-II and Ga-III. Again it makes clear that topological short range ordering in liquid gallium is more similar to both Ga-II and Ga-III than Ga-I within applied pressure range. This result is consistent with that of bond angle analyses reported by Yang *et al*. in the theoretical calculations^23^. In contrast, Yagafarov *et al*. proposed the local structure of liquid gallium is a mixture of Ga-I and Ga-II structures, has no information on the liquid density changed with pressure and the proof was quite indirect, since it was based on a fit of experimental PDFs by using known crystal structures of gallium^24^. Specially, an interesting phenomenon is that the profile of all peaks of angle distribution in Ga-II looks like a shifted that of liquid gallium.

The structural similarity between liquid Ga and both solid phase II and III may be related to the process of melting according to the free volume theory^33–35^. Melting is a result of increase in free volume in the solid system to some critical value, although the total molar volume of the system can either increase or decrease depending on the structural change^36, 37^. In general, in the process of melting, the crystalline phase expands and the total molar volume increases, so that sufficient free volume is presented to produce melting. The local atomic arrangement is thus related to its crystal form to some extent.

**Figure 5:**
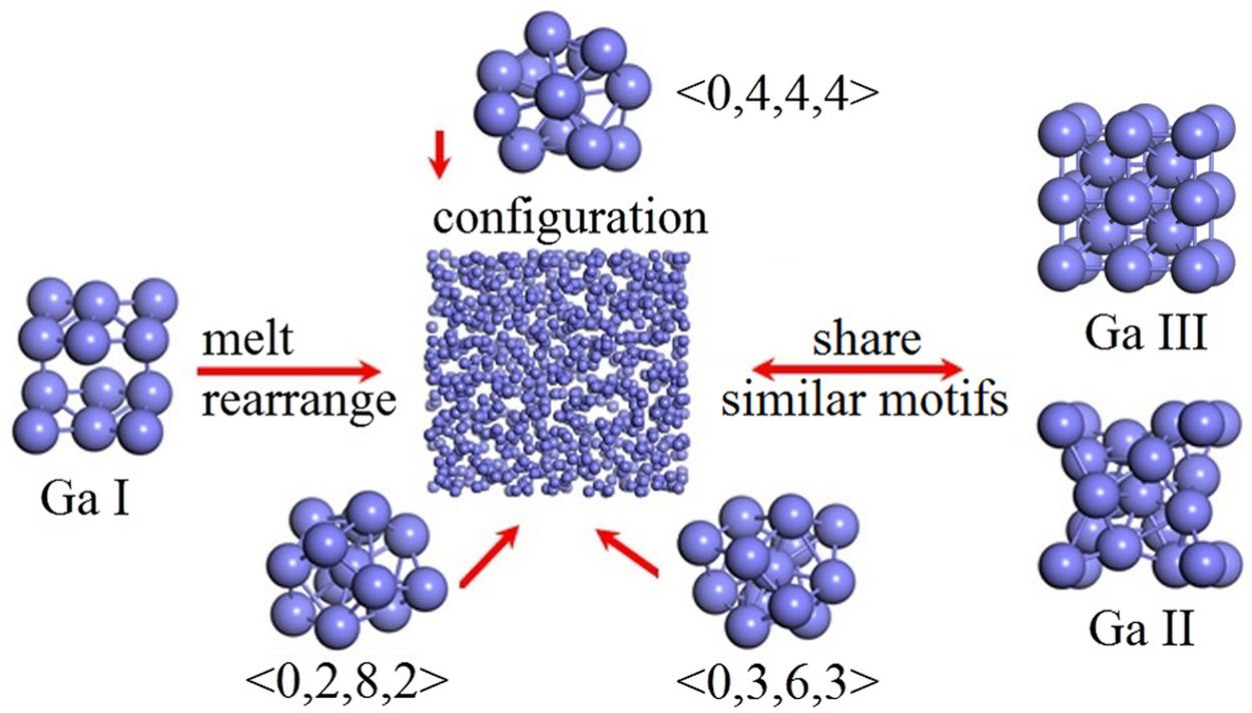
Schematic illustration of the melting of liquid gallium. Ga I rearrange to form locally denser Voronoi polyhedra to get sufficient free volume between the Voronoi polyhedra. Liquid gallium, Ga-II and Ga-III share the similar motifs.

However, the slope of melting curve of Ga-I is negative, and liquid gallium has a higher density when Ga-I melts, similar to the ice-water case. The unit cell of the crystalline phase Ga-I consists of eight atoms, two of which have a very close distance of ~2.44 Å, with the other six atoms, also in pairs, a little further away at 2.7–2.8 Å^38^, with an average coordination number ~7. The Ga_2_ pairs are dimer-like in this structure, which is significantly less closely packed than the liquid with a coordination number of 11–12, closing to that of 12 in both Ga-II and Ga-III. Hence, upon melting, the topological structure of Ga-I must break up and reconstruct. To achieve a high density and packing efficiency, the local atomic arrangements of liquid metal cannot be truly random but must meet both topological and chemical requirements^39^. Furthermore, the pressure-induced volume decrease is mainly caused by decrease in coordination spheres beyond the first coordination shell^16, 23^. The open packed phase I structure^38^, with an average CN~7, has significant amounts of free volume and contracts during melting, breaking the crystalline atomic configuration and forming locally denser motifs, such as the 3 typical Voronoi polyhedra as shown in Fig. 5, similar to those in both Ga-II and Ga-III. The quantities of free volume are located between locally denser motifs. The dimer-like Ga_2_ units largely disappear in the liquid state, because thermodynamically all Ga atoms are equal in the disordered state, although the second peaks around 3 Å^−1^ in Fig. 1(a) may suggest some residual dimer-like units in the melt. Thus the liquid structure is characterized by predominantly Ga-II like and Ga-III like motifs with some residual dimer-like motifs from the Ga-I phase^16, 23, 24^.

In summary, no detectable liquid-liquid phase transition is found in liquid gallium under high pressure up to 1.9 GPa at room temperature, based on *in situ* high energy X-ray PDF and microtomography measurements. The RMC simulations are used to characterize the local structure of liquid gallium, which more closely correlates to that of solid Ga-II and Ga-III. A melting process for gallium based on free volume theory is proposed. We hope that these findings will trigger further investigations into liquid gallium and other liquid materials with an ice-water type phase diagram.

## Methods

### High-pressure PDF

Total scattering data of liquid gallium at high pressure and ambient temperature were collected at the 11-ID-B beamline at the Advanced Photon Source at Argonne National Laboratory. A solid gallium sample with 99.9999% purity was heated to its liquid form and loaded into a 270 μm diameter hole in a T301 stainless steel gasket sandwiched between two diamond anvils. An annealed ruby ball was included as a pressure marker to quantify the pressure during the experiment^40^. The sample was compressed to 1.9 GPa at room temperature, and the experimental data points in a P-T diagram are shown in Fig. 1(a) (inset) as red hexagons. An amorphous silicon-based area detector with 2048 × 2048 pixels (unbinned) covering a 41 × 41 cm active area was used to collect X-ray scattering images. Images consisting of a summation of 150 5 s exposures provided an adequate signal-to-noise ratio at each pressure.

Raw image data were processed with the software Fit-2D^41^, applying a previously reported masking strategy^42^ to eliminate the structured contributions from the diamond scattering to the one-dimensional scattering data. Subtracting the contributions from the sample environment and background, the reduced PDF *G*(*r*) and structure factor *S*(*Q*) were extracted using the program PDFgetX2^43^, which performs a numerical Fourier transform of *S*(*Q*) according to

1 $$G(r)=4\pi r{\rho }_{0}(g(r)-1)=\frac{2}{\pi }{\int }_{0}^{\infty }Q[S(Q)-1]{\rm{s}}{\rm{i}}{\rm{n}}(Qr)dQ,$$

where *ρ*_*0*_ is the average atomic number density and *g*(*r*) is the pair distribution function. Compton scattering from the sample was corrected based on tabulated values for the known sample composition. The detector response was corrected using established geometric corrections using a transmission coefficient of 0.8 for the 0.5 mm detector phosphor at ~86.7 keV.

### RMC simulation under pressure

Since PDFs provide one-dimensional real space structure information, the RMC method was applied to generate three-dimensional atomic structure models consistent with the experimental data^3, 4^. The RMC simulations, using RMC++ ^44^, optimized atoms within a cubic box with periodic boundary conditions containing 12,000 atoms to fit the liquid gallium X-ray scattering data. The closest approach of pairs of atoms was constrained to be 2.30 Å. The number density as a function of pressure at ambient temperature for each configuration was based on X-ray microtomography measurements in which the isothermal bulk modulus was determined to be *B*_*0*_ = 23.6 GPa. The densities of liquid gallium at various pressure conditions are presented in Table 1, where the starting density, 6.097 g/cm^3^, is from the literature^45^. The methods used to evaluate the pressure induced volume changes are described in detail in our previous work^25^.

**Table 1:** The densities of liquid gallium at ambient temperature under various pressure conditions.

*P* (GPa)	0	0.3	0.6	0.8	1	1.3	1.6	1.9
*ρ* (g/cm^3^)	6.097	6.173	6.246	6.295	6.340	6.408	6.475	6.539
